# Iron metabolism in colorectal cancer

**DOI:** 10.3389/fonc.2023.1098501

**Published:** 2023-02-22

**Authors:** Luji Huang, Wangji Li, Yan Lu, Qinuo Ju, Manzhao Ouyang

**Affiliations:** ^1^ Department of Gastrointestinal Surgery, Shunde Hospital, Southern Medical University (The First People’s Hospital of Shunde Foshan), Foshan, Guangdong, China; ^2^ The Second School of Clinical Medicine, Southern Medical University, Guangzhou, Guangdong, China; ^3^ Good Clinical Practice (GCP) Center, Shunde Hospital, Southern Medical University (The First People’s Hospital of Shunde Foshan), Guangdong, China; ^4^ Guangdong Country Garden School, Shunde, Foshan, Guangdong, China

**Keywords:** iron metabolism, CRC, iron supplement therapy, CRC related genes, ferroptosis

## Abstract

Iron, as one of the essential trace elements in the human body, is involved in a wide range of critical biochemical reactions and physiological processes, including the maintenance of the normal cell cycle, mitochondrial function, nucleotide metabolism, and immune response. In this context, iron is naturally associated with cancer occurrence. Cellular iron deficiency can induce apoptosis, however, iron can also engage in potentially harmful reactions that produce free radicals because of its capacity to gain and lose electrons. Studies suggest that dietary iron, particularly heme iron, may be one of the leading causes of colorectal cancer (CRC). Moreover, patients with CRC have abnormal iron absorption, storage, utilization, and exportation. Therefore, iron is crucial for the development and progression of CRC. Elaborating on the alterations in iron metabolism during the onset and advancement of CRC would help to further explain the role and mechanism of iron inside the body. Thus, we reviewed the alterations in numerous iron metabolism-related molecules and their roles in CRC, which may provide new clues between iron metabolism and CRC.

## 1 Introduction

As an essential trace element, iron is associated with cell proliferation, enzymatic reactions, energy metabolism, cellular respiration, folate metabolism, DNA synthesis, and repair mechanism (1). Iron deficiency causes cellular G1/S arrest, affects cell proliferation, and promotes cell apoptosis. But excess intracellular free iron generates high levels of reactive oxygen species (ROS) through the Fenton reaction, damaging DNA, RNA, proteins, and lipids (2, 3), which can be mutagenic. CRC is inextricably linked to iron metabolism, iron overload due to various genetic mutations or excessive dietary iron intake is thought to be a major cause of CRC. And deregulation of the iron transportation system in the gut and increased iron content in tumor tissue can be observed in patients with CRC (4). Dysregulation of iron transporter (DMT1) is critical for HIF-2α mediated colon carcinogenesis as it increased tumor iron and exacerbated cell proliferation (5). It is widely accepted that red meat consumption increases the risk of CRC (6). In 2015, the International Agency for Research on Cancer classified the consumption of processed meat as carcinogenic and red meat as probably carcinogenic in humans (7). The TCGA database analysis revealed that iron homeostasis is deregulated in CRC patients. And patients with disrupted iron metabolism have a significantly worse outcome than those with normal iron metabolism (8). But what is the exact relationship between iron and CRC is still elusive. Thus, unveiling the change of iron metabolism in CRC is necessary. This review depicted the characteristics of iron metabolism in CRC by summarizing previous literature on the alterations of several molecules of iron metabolism in CRC and their possible mechanisms, hoping to elucidate the link between CRC and iron metabolism.

## 2 Iron

### 2.1 Normal iron metabolism

Dietary nonheme iron is reduced to the ferrous form in the gut lumen by gastric acid or ferrireductases such as duodenal cytochrome b reductase 1, which is located on the apical surface of enterocytes (9, 10). Second, ferrous iron is transported across the apical surface of enterocytes by divalent metal transporter 1 (DMT1) (SLC11A2). The amount of iron that adult men and women, on average, absorb from their diets is 6% and 13%, respectively (11). Unabsorbed iron passes through the digestive tract and gathers in the colorectum before being eliminated in the stool.

Apart from being utilized by enterocytes, a proportion also enters the portal and peripheral circulation *via* ferroportin (FPN) before it is converted to ferric iron by hephaestin or ceruloplasmin (12). Iron is transported to target tissues and organs (like bone marrow) by binding to transferrin (TF), a serum iron-binding protein produced largely by the liver, along with iron released from macrophages after senescent erythrocytes’ degradation and stored iron releasing from hepatocytes. Iron enters the endosomes *via* endocytosis as the transferrin-transferrin receptor 1 complex (TF-TfR1). And ferric iron is transformed to ferrous iron under the reduction of STEAPs and mediated by DMT1, forming the intracellular labile iron pool (ILP) (13), from which iron is delivered to multiple intracellular destinations. Iron is used in the synthesis of heme and iron-sulphur clusters, which are then incorporated into proteins that carry out the citric acid cycle, oxidative phosphorylation, and many other crucial functions. Iron is incorporated into the active site of proteins like ribonucleotide reductase, where it participates in the catalytic conversion of ribonucleotides to deoxyribonucleotides.

Excess iron is stored in iron storage protein ferritin (FT) or exported by FPN (14). Iron homeostasis is vital for normal physical function. And it is primarily achieved through the interaction of iron regulatory protein (IRP) and iron-responsive elements (IREs) on the cellular level, whereas systemic iron homeostasis is primarily regulated by hepatocytes secreted hepcidin, which binds to FPN and causes its degradation.

### 2.2 Impaired iron metabolism in CRC

Prussian blue staining indicated significant iron accumulation in colon cancer tissue compared to normal colon mucosa. And high iron diet feeding mice had a higher colon tumor burden, while low iron treatment reduces HIF-2a–induced colon tumor formation (5). Hereditary hemochromatosis (HH), characterized by iron overload, is primarily resulted from a substitution of a cysteine with a tyrosine at position 282 in the HFE protein (C282Y) (15). Homozygotes of the C282Y mutation had more than twice the risk of CRC than those with wild type (16). CRC growth is highly dependent on elevated intracellular iron (17). Many gene expressions involved in iron metabolism were abnormal in CRC, resulting in increased iron uptake and decreased iron export compared to normal colonocytes, culminating in tumor iron accumulation, accelerated cell proliferation, and repressed cell adhesion. The activated HIF-2α signaling pathway has been shown to induce colon cancer by increasing intracellular iron content (5). A diet rich in iron significantly increased colonic tumor burden in a mouse model of colitis-associated colon tumors (17). And it cooperated with colonic inflammation to activate the IL-6/IL-11-Stat3 signaling pathway to promote tumor formation (18). Increased iron levels in colonic epithelial cells following *APC* or *β-catenin* mutation promoted colon tumor formation by activating the Wnt signaling pathway (19). Reducing dietary iron intake delays the age of colon cancer onset in patients with hereditary non-polyposis colorectal cancer-associated mismatch repair gene mutations (20). Although increased dietary iron intake is thought to be associated with colorectal tumorigenesis, a case-control study of female found no significant association between body iron levels and colorectal adenomas. But dietary iron intake does not correspond with systematic iron level. And that iron level in the intestinal lumen is more tightly associated with colorectal tumorigenesis than circulating or tissue iron (21).

CRC cells require iron for growth and division, but too much iron is hazardous and can be cytotoxic and genotoxic, causing oxidative damage to cells. Intracellular ferrous iron induces cell death and organ dysfunction by causing significant ROS production, harming biological macromolecules such as proteins and nucleic acids, and damaging the cell membrane (22). In contrast to apoptosis, necrosis, and pyroptosis, this novel mechanism of cell death is known as ferroptosis, which is characterized by cellular iron excess and lipid peroxidation.

### 2.3 Heme and CRC

The risk of CRC is strongly associated with nutrition, especially with diets high in red meat (beef, pork, or lamb) that contains the iron-porphyrin pigment: heme. However, consumption of white meat (such as poultry and fish), which contains much less heme is not associated with an increased CRC risk (23, 24). This made a connection between CRC and heme consumption. Because heme is only moderately absorbed in the small intestine, the vast majority of heme (about 90%) reaches the colon as demonstrated in a feeding study in rats (25). Thus, the high heme load of the colon may be the cornerstone of its carcinogenesis.

Dietary heme is mostly absorbed in the proximal intestine, with absorptive capacity decreasing distally. A membrane protein named HCP 1 (heme carrier protein 1), with homology to bacterial metal-tetracycline transporters, mediates heme uptake by cells in a temperature-dependent and saturable manner. HCP 1 was localized to the brush-border membrane of duodenal enterocytes, and was regulated by iron and hypoxia (26). But later, HCP 1 was shown to serve as a high-affinity, pH-dependent folate transporter and renamed as proton-coupled folate transporter (27). The second involves the resorption of heme into enterocytes by receptor-mediated endocytosis, which seems to be mediated by a protein that is located on top of the microvilli, i.e. the apical membrane of the intestine, and binds heme to promote its internalization (28). Lastly, heme is cleaved by heme oxygenase-1 (HO-1), producing carbon monoxide (CO), iron ion, and biliverdin (29). CO and bilirubin derived from biliverdin are antioxidants which may benefit cells in growing and surviving. Thus, HO-1 is a cytoprotective enzyme (30–32).

There are many reasons that are responsible for the carcinogenic property of heme. Heme catalyses the formation of N-nitroso-compounds, which in turn results in the initiation of colorectal carcinogenesis. Dietary heme injures the surface epithelium which is compensated by crypt cell hyperproliferation and by inhibition of apoptosis, resulting in hyperplasia (33). It is understood that (heme-) iron catalyzes the Fenton’s reaction, which leads to ROS production, the produced hydroxyl radical attacks DNA *via* addition to the double bonds of DNA bases and by abstracting hydrogen atoms from the C-H bonds of the 2-deoxyribose sugar moiety, thereby generating oxidative DNA lesions such as 8Oxoguanine (8-OxoG) and thymine glycol, single-strand breaks and abasic sites (34, 35). Heme appears to induce the production of H_2_O_2_ by HO-1 in colonic epithelial cells, and the produced H_2_O_2_ participates in DNA damage, cell proliferation, apoptosis, and the production of inflammatory cytokines like IL-8. This may indicate that long-term intake of heme is possibly involved in carcinogenesis by inducing chronic inflammation (36). ZnPP, a HO-1 inhibitor, reduced HIF-1α expression in HCT-15 cells, and consequently inhibited hypoxia-mediated VEGF release. ZnPP inhibited HCT-15 cell proliferation, and reduced tumor growth in the HCT-15-induced tumor xenografts, thus, it may be a potential therapeutic agent against CRC (37). And Bastide et al. fund that fecal water from rats given hemoglobin was rich in aldehydes and was cytotoxic to normal cells, but not to premalignant cells (38). Thus, heme consumption may screen out these premalignant cells and promote the CRC development. In addition, heme-induced cell hyperproliferation is intimately linked to microbial dysbiosis, which is closely related to CRC. Heme-supplemented diets (0.9 mol/g diet) changed the makeup of the gut microbiota and decreased α-diversity (39). The hyperproliferation in the colon epithelium of heme-fed mice was eliminated after receiving broad-spectrum antibiotics, emphasizing the strong relationship between these alterations and the intestinal microbiota (40).

### 2.4 Iron, microbiota, and CRC

After iron consumption, only 15% of dietary iron is absorbed within the duodenum, with the remnant passing into the large intestine where it has the potential to be utilized by colonic bacteria (41). Parmanand et al. found that the growth of potentially pathogenic bacterial, such as Salmonella typhimurium and Escherichia coli, was significantly inhibited when cultured in an iron-deficient medium, however, probiotic bacterial such as Lactobacillus rhamnosus were unaffected (42). This phenomenon suggests a possible role of colonic iron in promoting the growth of pathogenic bacteria, but not of probiotic bacteria. It has been validated in anemic African children who were given iron-fortified biscuits that dietary iron contributes to gut dysbiosis, and suggests that increasing pathogenic and decreasing beneficial bacterial populations has the potential to contribute to disease through inducing gut inflammation (43). Constante et al. found that oral heme iron altered microbial populations, specifically decreasing butyrate-producing taxa, which ultimately was associated with a decrease in fecal butyrate levels. Thus, oral iron has the potential to worsen disease by reducing butyrate, which has anti-inflammatory and anti-cancerous properties (39). However, not all CRC derives from gut inflammatory and dysbiosis, further explorations of microbiota on iron metabolism and CRC are required.

## 3 Proteins related to iron metabolism in CRC

### 3.1 Transferrin

Fecal occult blood test (FOBTs) is commonly used for large-scale population screening of CRC. But Wang et al. suggested that the sensitivity of Hb combined with TF in fecal CRC screening was higher than that of Hb alone (44). Therefore, fecal TF is an indicator of CRC existence which may serve as an alternative for CRC on-site screening when combined with Hb. Both multivariate and univariate analyses revealed that preoperative serum TF level is a novel prognostic marker in stage I-III CRC patients. A lower serum TF level was associated with shorter overall survival (OS), relapse-free survival and cancer-specific survival (45). MiR-545, which is highly expressed in colon cancer cell lines (HT-29, LoVo, and HCT-116), inhibits ferroptosis by inhibiting TF expression, lowering intracellular iron accumulation, as well as reducing lipid peroxidation (46). Besides, another study indicated that both low and high transferrin saturation (TSAT) heralded a poor OS in stage II-III CRC patients (47). Moreover, TF may be served as an indicator of curative effect of chemotherapy. Ochiai et al. (48) found that lower increase in blood TF level after 48 hours treatment with FOLFOX or FOLFIRI chemotherapy regimens was linked to a shorter median survival time (MST). That is to say, serum iron may be a useful and convenient predictor of the response to chemotherapy. Patients with colon cancer liver metastases who have undergone partial hepatectomy have a poor prognosis if their serum TF levels are less than 190 mg/dL (49), which means serum transferrin level is a potential predictor of poor OS in patients with CRC liver metastases after hepatic resection. When it comes to CRC treatment, TF-binding peptide (TBP-Ps) functionalized polymers can selectively and stably bind to TF and deliver doxorubicin (DOX) to TfR-overexpressing HCT-116 cells (50), providing an alluring strategy in formulating TF-targeted nanomedicines. Transferrin-lipoplexes containing the therapeutic gene *IL12* inhibited tumor growth in CT26-derived tumor-bearing animals, resulting in complete tumor regression in 75% of the treated mice (51). Thus, the complex established an efficient targeted non-viral strategy for *IL12* gene transfer in colon cancer.

### 3.2 Transferrin receptor 1

**Figure 1:**
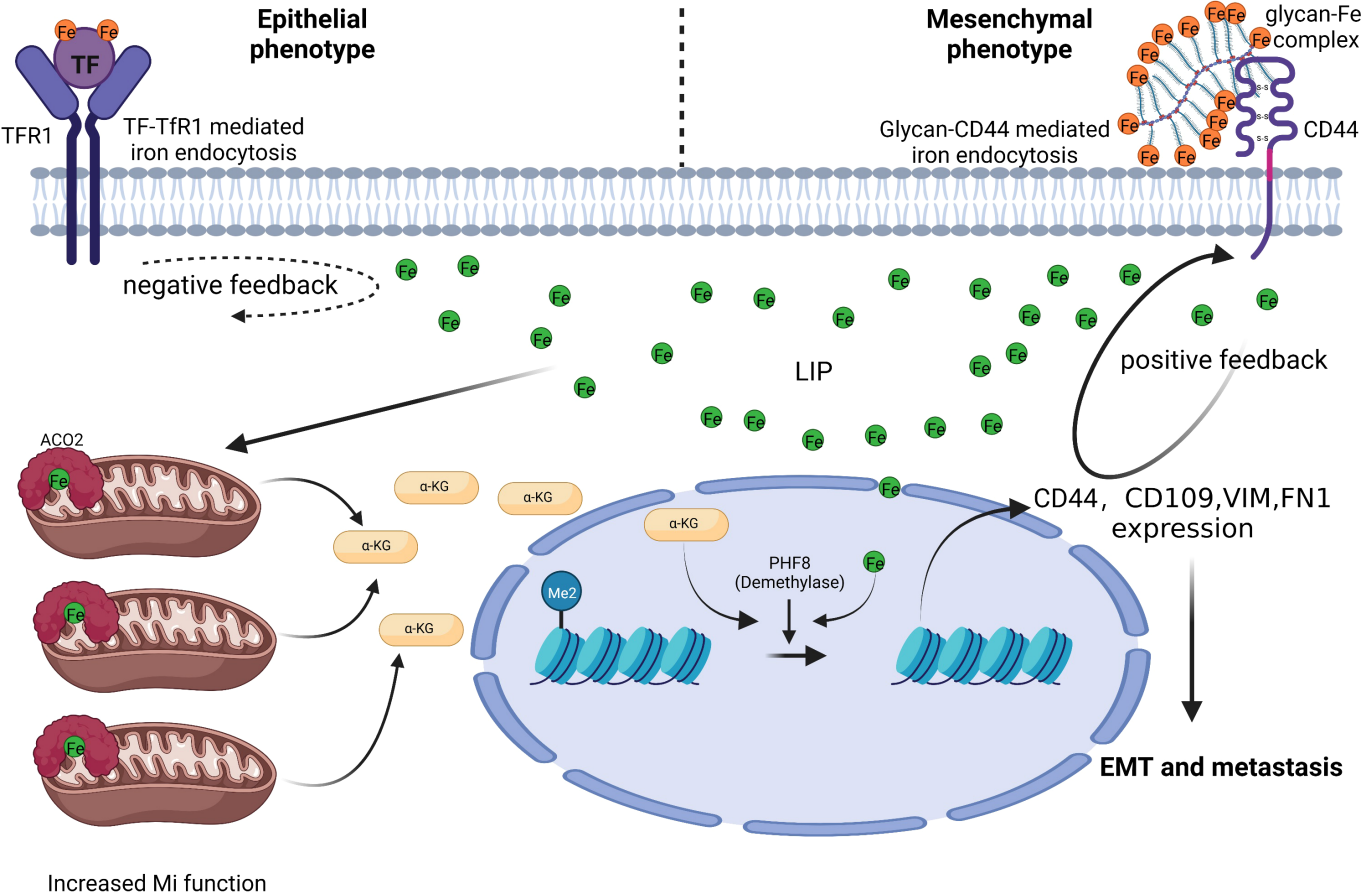
CD44 mediates iron absorption when CRC cells undergo EMT. CRC cells with an epithelial phenotype import iron *via* the TF-TfR1 mechanism. CRC cells with a mesenchymal phenotype need more iron, but the TF-TfR1 mechanism is limited by negative feedback between intracellular iron and TfR1. Glycan-Fe(III)-CD44 mediated iron absorption can aid CRC cells in sequestering iron and improve mitochondrial function and α-ketoglutaric acid(α-KG) production. Increased α-KG cooperates with ferrous iron to boost the plant homeodomain finger protein 8 demethylase activity. Ultimately, this causes EMT associated genes (including *CD44*) expression (58). Thus, positive feedback is formed between intracellular iron and CD44 to satisfy the higher iron need of mesenchymal phenotype CRC cells and facilitate CRC metastasis.

Colon cancer tissues have much lower level of TfR1 expression than nearby normal tissues, which prevents it from absorbing iron *via* TfR1 during colorectal carcinogenesis (8). TfR1 has a parallel relationship in individual blood and colon tissue, and it can be used as a potential marker for screening CRC and precancerous lesions together with adenosylhomocysteinase (SAHH) and immunoglobulin heavy variable 3-7 (HV307) (52). However, there is also study claimed that TfR1 mRNA and protein levels in colon cancer tissue are significantly higher (53). TFRC is induced by adenomatous polyposis coli (APC) gene loss-driven β-catenin activation in CRC, iron chelation, and TFRC disruption increase DNA replication stress, DNA damage response, apoptosis, and reduce colon tumor growth (54). Decreased expression of MiR-107 in colon cancer tissue prevented the proliferation, migration, and infiltration of CRC cell SW620 by inhibiting TfR1 expression (55). Although TfR1 was up-regulated in CRC, there is evidence that down-regulation of TfR1 promotes cancer progression. TfR1 down-regulation activates the JAK/STAT3 pathway, promotes the tumor cells transformation from G1 to S phase, and enhances cells motility and infiltration. The expression level of TfR1 decreases with the decrease of CRC cell differentiation, and CRC patients with high TfR1 expression have a better survival (53). TfR1 is highly expressed in Dukes grades A, B, and well-differentiated CRCs, while it is lowly expressed in Dukes grades C, D, and poorly differentiated, lymph node invasive, and distant metastases CRCs (56). This phenomenon indicates that the expression mode and role of TfR1 differ in different stages of CRC. In addition, Fumiyasu et al. found that the TfR1 expression level is affected by the circadian rhythm (57). And this expression mode can be a significant aspect of strategies for TfR1 targeted cancer therapy. α-AT deficiency is linked to colon carcinogenesis. It inhibits neutrophil elastase activity and tumor metastasis, and α-AT can prevent TF from binding to TfR1. Colon cancer invasion and metastasis were increased in α-AT deficient individuals, and TfR1 binding to TfR1 is not inhibited, resulting in TfR1 down-regulation (56). Another explanation is that when tumor cells undergo EMT, the demand for iron increases, which is accompanied by a shift in the way cells absorbing iron, from TfR1-mediated transferrin to CD44-mediated hyaluronic acid-iron complex as the main approach (**Figure 1**). CD44 forms a positive feedback regulation with intracellular iron, which differ from the negative feedback of TfR1, finally, increased intracellular iron inhibits TfR1 expression (58). This phenomenon partly explained why tumor cells get more iron without restricting by TfR1 down-regulation. And TF-linked liposomes encapsulated with oxaliplatin can be used for targeted delivery of chemotherapeutic drugs in colon cancers with high TfR1 expression to improve anti-tumor efficacy (57).

### 3.3 Divalent metal transporter 1

DMT1 is a transmembrane glycoprotein that mediates the transportation of divalent metal ions such as Fe^2+^, Zn^2+^, Mn^2+^, Co^2+^, Cd^2+^, Cu^2+^, Ni^2+^, and Pb^2+^ in a pH-dependent, proton-coupled manner. Whereas ferric iron must be reduced to ferrous iron before entering cell through DMT1 (9, 59). Because colonic epithelial cells are not involved in iron absorption, the DMT1 expression is extremely low (almost undetectable). However, DMT1 is up-regulated at both mRNA and protein levels in CRC tissues, leading to increased apical iron uptake, activated CDK1/JAK/STAT3 growth-promoting signaling axis. Reducing DMT1 expression significantly inhibited colon cancer proliferation (17). Certain DMT1 single nucleotide polymorphism also increases cellular iron uptake and are associated with colorectal carcinogenesis (60). Experiments on mice showed that HIF-2α-mediated induction of DMT1 expression and a subsequent increase in intracellular iron are critical in adenoma-adenocarcinoma transformation (5). Further research indicated that DMT1 over-expression appears to be a relatively late event in colorectal carcinogenesis, as is evidenced by the fact that DMT1 expression in low- and high-grade adenomas did not differ (61). Minor et al.

showed that colon tissue of ulcerative colitis (UC) significantly increased DMT1 expression (62). Thus, DMT1 may also contribute to the inflammatory process of UC and influence the occurrence of colitis-related CRC. Recently, Pyrimidinone 8, a compound that inhibits DMT1 activity, was discovered. It inhibits cellular iron uptake in a pH-independent manner while not changing DMT1 expression and may provide the basis for future antitumor drugs targeting DMT1 (63).

### 3.4 Ferritin

Ferritin is a protein composed of 24 subunits, within two types of subunits: ferritin light chain (FTL) and ferritin heavy chain (FTH). Cao et al. revealed that colorectal adenomas are not linked with systematic iron metabolism markers (ferritin, TF, and TF saturation) (21). Moreover, ferritin level in colon cancer tissue is identical to that in healthy colon tissue. There are no differences in ferritin levels between CRC and adenoma patients and individuals with a normal colonoscopy. Therefore, screening for CRC with the body ferritin level seems to be impossible. However, high blood ferritin level has also been linked to an increased incidence of colon adenomas (64), with the relationship being stronger in the right hemicolon (65). This implies that left hemicolon and right hemicolon may have distinct iron metabolism model. Given that patients with serum ferritin (SF) level below 100 ng/mL are nearly five times more likely to develop colonic neoplasms than those above 100 ng/mL, Sawhney, et al. recommended these individuals should undergo colonoscopies (66). In a prospective study of patients with involuntary weight loss, ferritin level greater than 100 ng/mL are considered to rule out colon cancer but not gastric cancer or rectal cancer (67). This suggests that the SF still has some predictive value for the occurrence of colon cancer, and it picks out the high-risk groups for further examination like colonoscopies. Because FTH also binds to TfR1, targeting of CRC using FTH encapsulation of chemotherapeutic drugs is considered a relatively efficient and secure method (53). For example, apoferritin can be used to transport chemotherapeutic drugs as a novel nanoparticle with broad clinical application potential. Lin et al. demonstrated that the combination of oxaliplatin-coated apoferritin nanoparticles and pembrolizumab could significantly inhibit the high EGFR expressing CRC cell growth and prolong the survival of tumor-bearing mice (68). In addition, Xiong et al. discovered that copper polypyridine compounds encapsulated in apoferritin nanoparticles significantly restrained the development of multidrug-resistant CRC by inducing autophagy-dependent apoptosis (69). Thus, the value of clinical application of apoferritin deserves further exploration. On the other hand, the expression of FTL in colon cancer increased, which can modulate the sensitivity of colon cancer to chemotherapy. According to Li et al. FTL is up-regulated *via* the Linc00467/miR-133b pathway, resulting in cell resistance to 5-FU and promoting colon cancer metastasis. FTL levels in cancer tissue, tumor cells, and serum were negatively correlated with patient survival (70). Therefore, serum FTL level appears to be a useful biomarker for selecting treatment regimens and monitoring tumor progression.

### 3.5 Hepcidin

**Figure 2:**
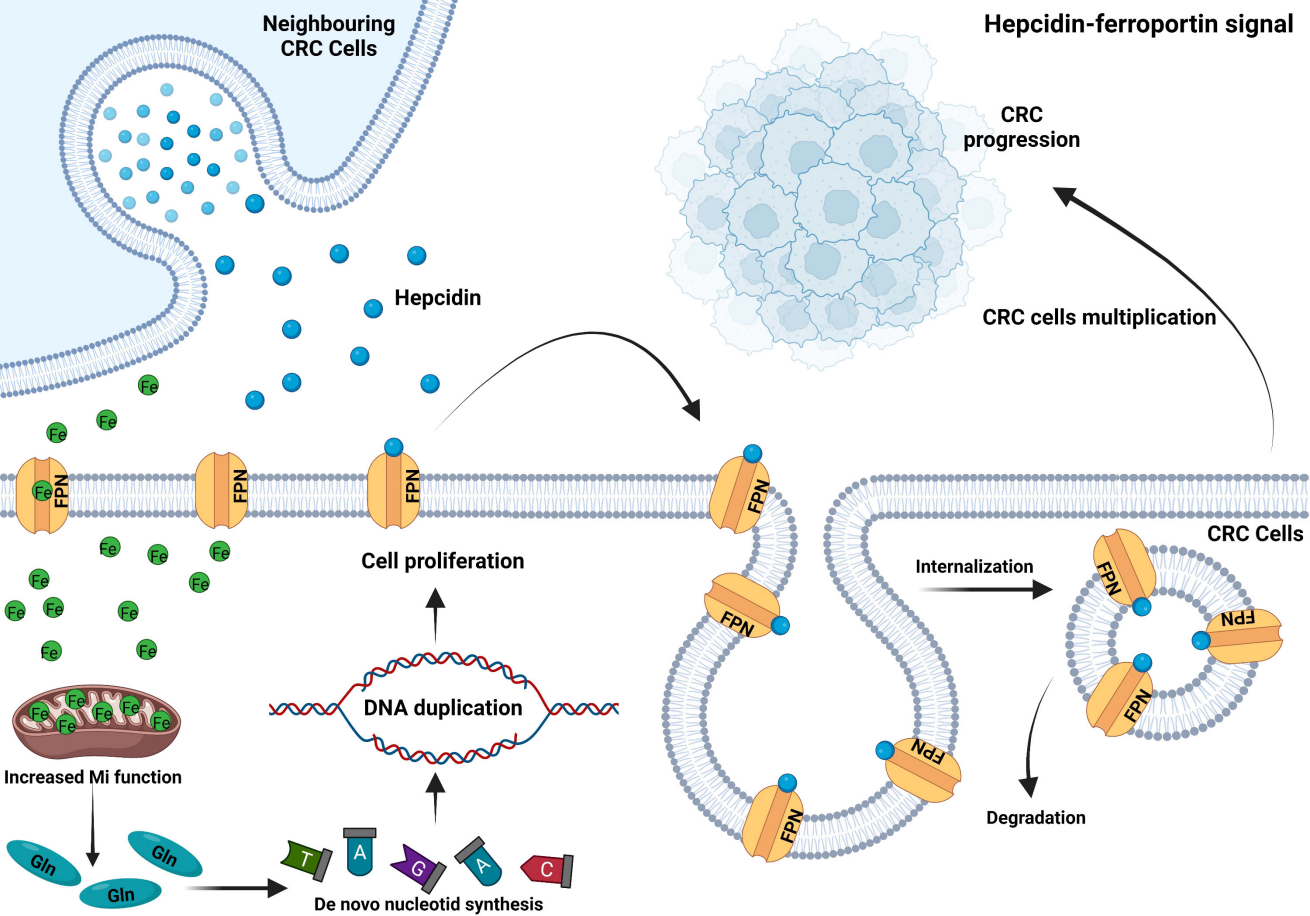
CRC ectopically expresses hepcidin to sequester iron. CRC cells secrete hepcidin in an autocrine or paracrine manner. Hepcidin binds to ferroportin on the plasma membrane, inducing its internalization and degradation. Thus, reduced iron exportat *via* FPN and sustained mitochondrial function to enhance glutamine production. Increased intracellular glutamine accelerates *de novo* nucleotide synthesis and DNA duplication, which leads to CRC cell proliferation and tumor progression (76).

Circulating hepcidin is a peptide hormone secreted by hepatocytes under normal conditions. It promotes ferroportin internalization and degradation and regulates the systematic iron homeostasis. Several proteins on the cell surface of hepatocytes are required to regulate hepcidin expression in response to bone morphogenetic protein (BMPs) as well as diferric transferrin. These factors include homeostatic iron regulator (HFE); hemojuvelin (HJV); transferrin receptor 2 (TFR2); BMP receptor proteins ALK2, ALK3, and BMPR-II; neogenin; and TMPRSS6 (71, 72). Hepcidin deficicency causes iron overload, while excess causes hypoferremia and anemia. Normal colon tissue does not express hepcidin, but approximately 46% of colon cancer cells express hepcidin in the membrane and cytoplasm (73). Phillips et al. found that hepcidin could improve the survival and proliferation of CRC cell SW480 after co-culture (74). Furthermore, hepcidin in patients’ urine and tumor tissue correlated positively with CRC T staging (73, 75). It has been discovered to be abnormally activated in CRC (**Figure 2**) (76), and maintained tumor cells’ nucleotide metabolism and mitochondrial function by increasing iron level (77). However, in the mouse colon cancer model lacking ectopic hepcidin expression, tumor number, size, and burden reduced, indicating that hepcidin ectopic expression in CRC facilitated tumor development. A survival analysis of 530 colon cancer patients revealed that those with high hepcidin expression in tumor tissue had considerably shorter survival than those with low hepcidin expression (77). The ability of colonic epithelial cell to secrete hepcidin can be enhanced by IL-6 and leptin (74), which may explain why obese people have a higher incidence of CRC than the general population.

### 3.6 Lipocalin2

Lipocalin2 (also known as neutrophil gelatinase-associated lipocalin (NGAL)) is a protein secreted by innate immune cells such as macrophages, hepatocytes, and fibroblasts in response to pathogens’ stimulation of Toll-like receptors. It can compete with the pathogen siderophores to bind iron, limiting iron uptake by pathogens, inhibiting their growth, and achieving the effect of the innate immune response (78). In addition, lipocalin2 can be produced by gastrointestinal epithelial cells and aids in epithelium regeneration and repair and maintaining mucosal integrity (79). CRC development and lipocalin2 have recently been discovered to be intricately linked. Lipocalin2 regulates intracellular iron in a bidirectional manner, promoting both iron uptake and excretion and changing the relative content of ferric and ferrous iron. And it is substantially up-regulated in colon cancer tissue compared to adjacent normal colon tissue (80). Higher lipocalin2 level in colon cancer tissue is associated with cancer progression, advanced tumor stage, and poor patient prognosis (81). Although its levels in patients’ serum and colon cancer tissue are both above normal ranges, lipocalin2 expression vary among CRC cell lines. For instance, SW620 and RKO cell lines scarcely express lipocalin2 while SW480, HCT-116, LoVo, CW-2, HT-29, and LS513 cell lines highly express lipocalin2. Since most CRCs are adenoma-adenocarcinoma developmental patterns, exploring the expression of lipocalin2 during adenoma-adenocarcinoma transformation helps to unveil the role of this molecule in the occurrence and progression of CRC. Lipocalin2 is highly expressed in most colorectal neoplastic lesions, and this occurred during normal epithelial-adenoma transformation but not during adenoma-adenocarcinoma transformation, indicating that lipocalin2 overexpression in colorectal carcinogenesis is a relatively early event. Thus, serum lipocalin2 cannot be served as a sensitive and effective marker for colorectal adenoma malignant transformation (82, 83). In addition, lipocalin2 can covalently bind to extracellular MMP-9 (matrix metalloproteinase 9), preventing its degradation and promoting tumor infiltration and metastasis by maintaining its activity. Furthermore, Zhang et al. found that rectal cancer tissue expresses higher lipocalin2-mRNA and MMP-9-mRNA, and the two are significantly correlated. Therefore, lipocalin2 may promote rectal tumor progression *via* MMP-9. Meanwhile, the high lipocalin2-mRNA expression is significantly related to tumor invasion depth, lymph node metastasis, venous invasion, and later pTNM staging (84). To our surprise, lipocalin2 can also promote 5-FU resistance in CRC cells (80, 81). It may be a therapeutic target for advanced CRC because the interaction of lipocalin2-integrinβ3 enhances the stability of integrinβ3, recruits SRC to the cell membrane for self-activation, activates the downstream AKT/ERK cascade, and ultimately activates the anti-apoptotic program. Although colon cancer expresses higher lipocalin2 than normal colon tissue, metastatic or advanced CRC expresses less than non-metastatic. When lipocalin2 was knocked out, tumor shifted from an epithelial to a mesenchymal phenotype, indicating that down-regulating lipocalin2 can promote EMT (85). Feng et al. also indicated that lipocalin2 may be an important negative regulator in EMT, invasion and metastasis of CRC *via* acting as upstream of NF-κB/snail signaling pathway, and combinative manipulation of lipocalin2 and NF-κB/snail pathway may represent a novel and promising therapeutic approach for CRC (86). Lipocalin2 has been shown to curb the ability of colon cancer cell line KM12SM (colon cancer liver metastasis cell line) to metastasize to the liver. And knocking out lipocalin2 in HCT-8 CRC cell induces tumor cell resistant to vincristine. These findings imply that lipocalin2 plays different roles in different cell lines and these effects can not be applied universally (87). Because lipocalin2 regulates iron metabolism bilaterally, tumor cells can use it to uptake more iron to meet their metabolic needs while also excreting iron from the cytoplasm in the form of the Fe-lipocalin2 complex. RNA sequencing of tumor cells isolated from the cerebrospinal fluid of patients with brain metastases of breast cancer and non-small cell lung cancer revealed that they over-expressed lipocalin2 and its receptor (SLC22A17). This accelerated the uptake of iron by tumor cells to meet the needs of their growth and proliferation (88). Further study is required to determine whether CRC cells in distant metastatic lesions also utilize this pattern for survival advantage.

### 3.7 Ferroportin

FPN is the only protein known to date to mediate iron export. It is expressed on the surface of intestinal absorptive cells, hepatocytes, macrophages, and placental cells and is responsible for exporting intracellular ferrous iron to plasma (89). As stated previously, hepcidin directly targets FPN, promotes its internalization and degradation. And improper activation of the hepcidin-FPN regulatory axis is one of the major features of CRC that aids in iron accumulation. Increasing intracellular iron levels by depleting FPN on the cell membrane increased tumor size, number, and burden (77). Although FPN is up-regulated in non-advanced colorectal tumors, its cellular location is cytoplasm rather than the cell membrane, named “non-functional” FPN. Whereas FPN is down-regulated in advanced colorectal tumors (90), however, only male patients experienced this shift (61). Similar to TfR1, FPN may play different roles in different CRC stags. This suggests that both FPN expression and changes in subcellular localization influence tumor development.

### 3.8 Iron regulatory protein

**Figure 3:**
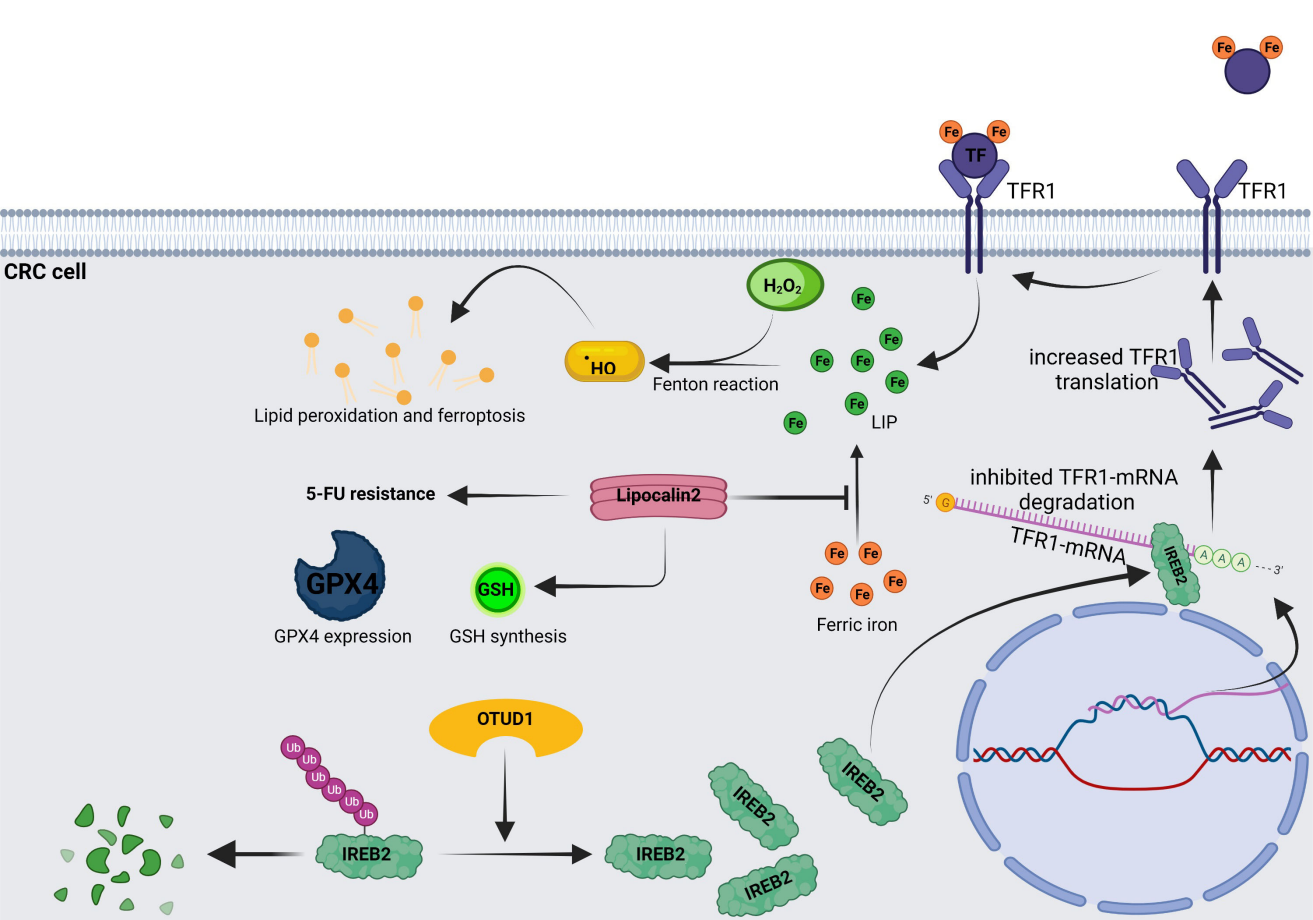
OTUD1 increases intracellular iron and induces ferroptosis in an IREB2-dependent manner. OTUD1 can increase IREB2 by deubiquitinating the IREB2-Ubs. Increased IREB2 binds to the IRE of TfR1-mRNA, which is located on the 3’-UTR, preventing its degradation and accelerating TfR1 translation (8). Increased TfR1 leads to mounting intracellular iron and subsequent lipid peroxidation and ferroptosis. Lipocalin2 can prevent ferric iron from transforming into ferrous iron, promote glutathione peroxidase4 (GPX4) expression and glutathione (GSH) synthesis, and inhibit lipid peroxidation and ferroptosis, which are responsible for the 5-FU resistance of CRC cells (80).

The iron-responsive element (IRE)/iron regulatory protein (IRP, IREB) system is primarily responsible for intracellular iron homeostasis. IRPs regulate the expression of several mRNAs by attaching to the iron response elements (IREs) on the 3’-UTR of these mRNAs (91). Although the expression of various proteins involved in iron metabolism has already been determined genetically, the IRE/IRP system that regulates the expression of these proteins at the post-transcriptional level continues to play a significant role. The IRE/IRP system regulates the expression of proteins involved in iron uptake (TfR1 and DMT1), storage (FTH and FTL), utilization (ACO2, SDH, and HIF2), and excretion (FPN). When IRP binds to the 5’-UTR of mRNAs (FTH, FTL, FPN, ALAS2, and ASO2), inhibits their translation, and when it binds to the 3’-UTR of mRNAs, inhibits their degradation (92). IRP has two isoforms, IRP1 and IRP2, with IRP2 being the primary regulator of iron. According to Horniblow et al., IRP2 expression increased during the development of CRC and was more remarkable in the right hemicolon cancer than in the left. Interfering with IRP2 offers a new therapeutic clue because the BRAF/MEK/ERK pathway is responsible for controlling IRP2 expression in CRC (93). Recently, study showed that OTUD1 was a colon-specific deubiquitinase that expressed in normal colonic epithelium goblet cells. According to studies on mice, maintaining OTUD1 function is critical for preserving normal intestinal iron absorption. In contrast, OTUD1 is specifically down-regulated in CRC, resulting in hyperubiquitination of its downstream protein, IRP2, promoting its degradation, and reducing iron uptake by CRC cells *via* TfR1 (**Figure 3**) (8).

## 4 Genes associated with CRC

Zhuang et al. found a total of 44 mutant genes with a mutation frequency higher than 5% in CRC in the TCGA population and validation patients. Among these, mutations in *TP53*, *APC*, *KRAS*, *BRAF*, and *ATM* covered 97.55% of the TCGA population and 83.02% of the validated CRC patients, which were shown to be associated with the development of CRC and can be used as diagnostic features (94). Some of these genes are related to iron metabolism in the process of CRC occurrence.

### 4.1 TP53

**Figure 4:**
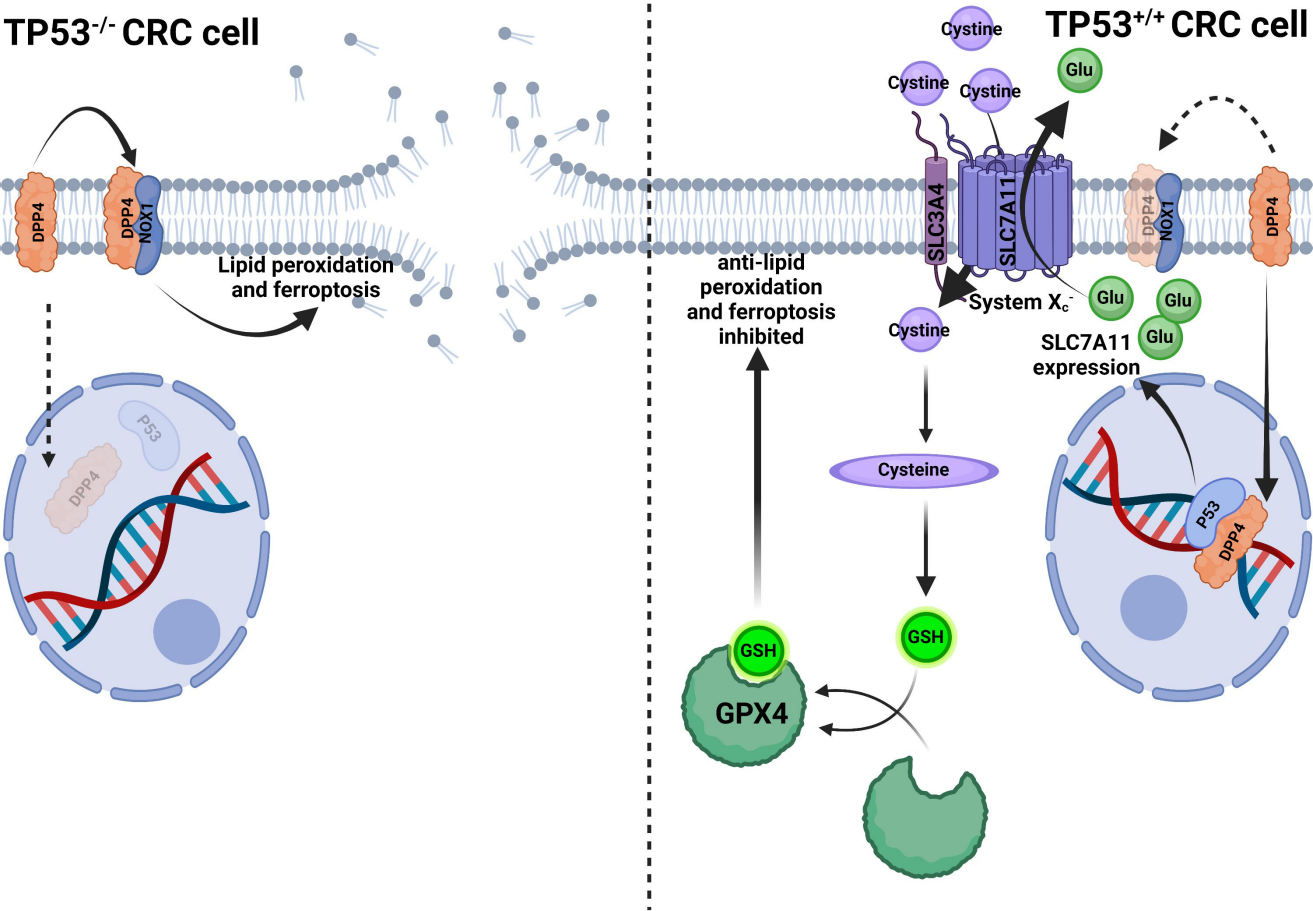
P53 inhibits ferroptosis in CRC cells. In *TP53^-/-^* CRC cells, DPP4 could not translocate into the nucleus to bind to P53 but remains in the plasma membrane and contacts with NOX1 to induce lipid peroxidation and ferroptosis. In *TP53^+/+^* CRC cells, DPP4 translocated into the nucleus to bind P53, facilitating *SLC7A11* transcription and enhanced ${X_{\text{C}}}^{-}$ function, thus, transferring more cystine into the cytoplasm and increasing glutathione (GSH) synthesis. Increased GSH enhanced GPX4 activity to compete with lipid peroxidation and ferroptosis (100).

The P53 protein, which is encoded by the tumor suppressor gene *TP53*, controls several cellular processes, including cell division, senescence, apoptosis, ferroptosis, DNA repair, angiogenesis, and autophagy. The P53 protein inhibits the cell cycle, triggers apoptosis, and integrates several metabolic pathways to achieve an anti-tumor effect. About half of all tumors have mutant *TP53*, which is the highest mutation frequency in different types of cancer (95). *TP53* is also related to CRC, its mutation directly causes colonic adenoma carcinogenesis, and is related to CRC metastasis. Tumor lymph node dissemination is driven by mutant *TP53*, regardless of whether containing wild-type *TP53*. However, tumor vascular invasion is caused by the loss of wild-type *TP53*, regardless of the presence or absence of mutant *TP53* (96). This seems to be the evidence that wild-type and mutant TP53 control different processes of CRC. Meanwhile, iron metabolism is intimately connected to *TP53*. Excess iron suppresses *TP53* expression, whereas iron deficiency promotes P53 accumulation. Intracellular heme can bind with P53 to suppress its transcriptional activity. P53 prevents iron overload at the cellular and systematic levels through different mechanisms (97). By-products of heme metabolism may induce *TP53* mutation and promote tumorigenesis (98). This indicates that iron and *TP53* interact with each other, and it may be the reason that red meat consumption increases CRC risk. Moreover, P53 induces apoptosis and inhibits the expression of *SLC7A11* at the transcriptional level, reducing cystine uptake and subsequent GSH synthesis to promote ferroptosis (99). However, the relationship between P53 and ferroptosis varies depending on the cell. For example, in human osteosarcoma and breast cancer cells (U2OS and MCF7), P53 promotes ferroptosis; on the contrary, in CRC cell lines (HCT116 and SW48), P53 inhibits ferroptosis by up-regulating the SLC7A11 expression (99, 100). In two CRC cancer cell lines (SW48 and HCT116), it was found that *TP53* mutation can enhance the sensitivity of CRC cells to the ferroptosis inducer erastin, and P53 can also alter the distribution of DPP4 in the cell membrane with a transcription-independent manner (**Figure 4**) to inhibit CRC ferroptosis (100).

### 4.2 APC

The tumor suppressor gene adenomas polyposis coli (*APC*), is mutated in familial and sporadic colorectal tumors. APC protein acts as a tumor suppressor effect by binding to a key molecule in the Wnt signaling pathway, β-catenin, and promoting its degradation (101). Dietary iron may affect the development of CRC by affecting the *APC* expression. Patients with CRC who had *APC* mutation and promoter 1A methylation consumed more processed and red meat than those with normal *APC* gene expression (102). Aberrant *APC* expression can synergize with iron to promote carcinogenesis. The intestinal epithelial malignant transformation can be triggered by a combination of iron in the lumen and the mutant *APC* (103). Increased intracellular iron in CRC cells with *APC* mutations (such as HCT116) can activate the Wnt signaling pathway without affecting the wild-type cells. DMT1-mediated intracellular iron increase after activation of HIF-2α, promoted colon carcinogenesis in *APC^min/+^* mice (5). Because 4HNE is significantly more toxic to wild-type than *APC* mutant colon cells, *APC* mutant cells can be selected after increased heme iron uptake to facilitate colorectal epithelial carcinogenesis (104).

### 4.3 KRAS

The GTP/GDP-binding protein encoded by the *KRAS* gene belongs to the RAS family of GTPases. After binding to GTP, KRAS activates multiple downstream pathways, such as RAF-MEK-ERK and PI3K-AKT-mTOR, promoting cell proliferation. While binding to GDP, KRAS is inactive. But after *KRAS* mutation, its function of hydrolyzing GTP is lost, and KRAS binds to GTP persistently and continuously stimulates cell proliferation (105). *KRAS* missense activating mutation was found in approximately 40% of CRC patients, with the vast majority occurring at codons 12, 13, and 61 (106). Heme iron consumption and CRC risk are linked to activating G > A mutations in *KRAS* and overall G > A mutations in *APC*. Other studies also confirmed that after red meat consumption, the heme iron metabolite N-nitroso compounds can induce the G > A mutation of *KRAS* (107). *KRAS* mutant CRC patients with low intratumoral FTH expression had longer three- and five-year survival than those with high expression, but not in wild-type *KRAS*. This suggests that the effects of FTH on CRC may be affected by aberrant *KRAS* expression. In addition, a starvation diet combined with ascorbate promotes ROS production in an iron-dependent manner, synergizing with chemotherapeutic drugs to improve efficacy against *KRAS* mutant CRC cells (108).

### 4.4 c-MYC

The proto-oncogene *c-MYC* is involved in cell proliferation, differentiation, and apoptosis. Abnormal *c-MYC* gene structure or expression mediates tumorigenesis through various mechanisms (109). *c-MYC* also regulates cellular iron homeostasis; c-MYC-induced cell proliferation and cell transformation depend on the inhibition of intracellular FTH expression (110). And it can up-regulate IRP2, rhythmically activate the *TfR1* transcription (57), increasing the intracellular iron pool and thus, promoting cell proliferation. TfR1 expression was reduced by nearly one-third in the *c-MYC* and *APC* double knockout compared to the *APC* knockout mouse model. This implies that *c-MYC* may collaborate with other genes to regulate iron metabolism, consistent with the increased iron demanding during cell proliferation (103). And it is also in line with the involvement of multiple (rather than single) genetic events in the pathogenesis of CRC.

## 5 Iron supplement therapy

There are 60% of CRC patients are anemic, and about 80% of anemic CRC patients had some forms of iron deficiency, with functional iron deficiency (FID) more prevalent than absolute iron deficiency (AID) (9.6% to 5.2%). Anemia in CRC patients is associated with poorer patient prognosis and postoperative complications; thus, iron therapy is necessary to correct anemia peri-operatively (111). Iron is necessary for appropriate immunological functions; hence, iron deficiency may hinder cancer immunosurveillance and potentially modify the tumor immune microenvironment, both of which may assist cancer development, thus, adequate iron therapy is needed to reduce these outcomes (112, 113). Iron supplementation is recommended in AID, but it is only recommended for symptomatic patients who have low SF in FID. And because of their poor duodenum iron absorption, intravenous iron (IVI) supplementation may be a superior therapy (114). IVI is more efficacious at improving quality of life than oral iron (OI) in anemic CRC patients (115). Currently, OI is the most used method of treating anemia in CRC patients, however, oral iron has the potential to boost procarciongenic bacterial populations in addition to a number of gastrointestinal side effects such abdominal discomfort, dyspepsia, and diarrhea (116, 117). Another study also indicated treatment with OI increased the serum proinflammatory cytokines like IL-6 and IL-17 (118), and IL-6 enhanced hepcidin expression (119), ultimately causing a vicious circle (as described previously, too much hepcidin leads to anemia) (77). OI supplementation results in an excess of biologically available iron to the colonic mucosa. However, iron is mainly distributed to stroma rather than epithelial cells in IVI, indicating that iron is less bioavailable to tumor cells (120). Consequently, to reduce the potential side effects and microbiota perturbation of iron supplements, a proper management should be implemented in anemic CRC patients.

## 6 Conclusion and discussion

According to Xia et al., there will be over 590,000 new CRC cases and about 300,000 deaths in 2022, and CRC will be the second most common malignant tumor in China (121). Red meat, processed meat, and heme iron consumption are associated with lung, breast, pancreatic, prostate, and colon cancer (122–124). Mounting dietary iron rather than systematic iron is critical for colon cancer development. This may be due to the direct exposure of the colorectal epithelium to the iron load of the colorectal lumen, which most tumors lack.

However, the results from studies assessing dietary iron intake and CRC risk were controversial (125, 126). These differences between studies may due to the complex interaction between foods within the digestive tract as well as the varied sources of iron within the diet. Many sources of dietary iron are healthy (fruit juice, and fortified cereals) thus, it is important to examine the sources of iron, for example, to distinguish between heme and nonheme iron (126).

We conclude that CRC patients have abnormal iron metabolism, and defective iron metabolism affects CRC incidence. Iron promotes CRC formation by enhancing the mitochondrial function, but also leads to ferroptosis while abundant. Although numerous indicators of iron metabolism in CRC patients have changed, more studies are needed to confirm their efficacy as markers for CRC detection, stages, and prognosis. Illustrating the precise mechanism of aberrant iron metabolism in CRC provides novel methods for CRC eradicating. Activating ferroptosis is another promising approach to improving the outcomes of targeted therapy and immunotherapy. For CRC patients with iron deficiency anemia, intravenous iron supplementation is more effective than oral iron supplementation (115, 120, 127), which does not increase the intestinal iron load, alter colonic flora, inflammation, and tumor progression (128). Therefore, choosing a desirable iron supplement to handle anemia and evade side effects is also worth to considering.

## Author contributions

All authors contributed to the review conception and design. Material preparation was done by YL, data collection was finished by WL, and analysis were performed by QJ. The first draft of the manuscript was written by LH, MO critically revised the draft, WL created the figures. And all authors commented on previous versions of the manuscript. All authors contributed to the article and approved the submitted version.
